# Kinesiophobia and Its Correlation with Upper Limb and Hand Functionality Among Individuals with Wrist/Hand Injury: A Cross-Sectional Study

**DOI:** 10.3390/jcm13247604

**Published:** 2024-12-13

**Authors:** Atenea Villalobos-García, Leire Cruz-Gambero, Roberto Ucero-Lozano, Kristin Valdes, Raquel Cantero-Téllez

**Affiliations:** 1 Tecan Hand Center, 29002 Málaga, Spain; atevillagar@uma.es; 2 Physiotherapy Department, IBIMA Hand Research Group FE-17, Faculty of Health Sciences, University of Malaga, C/Arquitecto Francisco Peñalosa (Ampliación Campus Teatinos), 29010 Málaga, Spain; leiregam@uma.es (L.C.-G.); cantero@uma.es (R.C.-T.); 3 Department of Physiotherapy, Faculty of Medicine, Health and Sports, European University of Madrid, 28670 Madrid, Spain; 4 InHeFis Research Group, Instituto Asturiano de Investigación Sanitaria (ISPA), 33011 Oviedo, Spain; 5 School of Occupational Therapy, Touro University Nevada, Henderson, NV 89014, USA; kvaldes2@touro.edu

**Keywords:** kinesiophobia, hand injury, pain catastrophizing, upper limb function

## Abstract

**Background/Objectives**: Wrist/hand injury incidences in the general population are high and contribute to a significant health problem. Fear of pain from movement can impact physical recovery, contributing to prolonged disability and impaired function in an upper limb after wrist/hand injury. The study’s objectives are (1) to evaluate the relationship between kinesiophobia, pain catastrophizing, *Quick*DASH, and Patient-Rated Wrist Evaluation and (2) to evaluate the data regarding the influence that basal kinesiophobia may have on upper limb functionality after wrist/hand immobilization. **Methods**: Participants referred from different medical centers with a wrist or hand injury that required immobilization were enrolled in the study. Data were collected just after the post-immobilization period. The following outcome measures were evaluated: the *Quick*DASH, the PRWE (Patient-Rated Wrist Evaluation), the TSK (Tampa Scale of Kinesiophobia), and the PCS (Pain Catastrophizing Scale). Demographics were summarized with descriptive statistics and linear relationships between variables using Pearson’s correlation coefficient. Furthermore, multivariate linear regression analysis was performed to determine whether kinesiophobia could predict upper functional performance. **Results**: 64 patients (40 women, 24 men) participated in the study. Significant kinesiophobia positive correlations were found between the TSK and the *Quick*DASH (*r* = 0.848, *p* < 0.001) as well as the TSK and the PCS error (*r* = 0.521, *p* < 0.001). The regression model explains 30.4% of the variance in upper limb function, suggesting that the PRWE, the Pain Catastrophizing Scale, and the *Quick*DASH are important in predicting dysfunction. **Conclusions**: Kinesiophobia may contribute to but is not a significant predictor of dysfunction in this model.

## 1. Introduction

Mechanical sciences consider the human body as a complex biomechanical system [1].

Clinical symptoms such pain or decreased range of motion that are present after a wrist/hand injury that requires immobilization can lead to a significant functional decline that can persist for 12 weeks following an injury [2]. Research on psychological factors indicates that fear of movement (Kinesiophobia) can reduce functional status and increase pain catastrophizing [3]. Kinesiophobia may also be directly associated with greater pain intensity and disability after musculoskeletal shoulder pathology [4,5].

Psychological factors can significantly influence the development of chronic health disorders, affecting proprioceptive function, muscle strength, and functional ability [6]. These variables also account for much of the variability in the disability observed among individuals with similar levels of impairment or injury. Evidence suggests that thoughts and emotions related to symptoms are closely linked to levels of comfort and functional ability in the context of a given injury or disease [7].

Recognizing the influence of psychological factors and maladaptive beliefs on pain perception during movement presents a modifiable target that can be leveraged to enhance treatment adherence and improve clinical outcomes in patients after wrist/hand injury. During recovery from upper extremity injury, patients sometimes express concerns regarding pain associated with increased use of the uninjured limb [8]. Factors such as self-efficacy, realistic expectations, confidence, and overall psychological readiness are linked to more favorable recovery outcomes, while kinesiophobia and unrealistic expectations are associated with poorer recovery trajectories [9].

Kinesiophobia is characterized as an excessive and irrational fear of movement and physical activity, stemming from the belief that such actions will lead to injury or reinjury [10]. Individuals with kinesiophobia often avoid or restrict their movements, overestimating the risk of injury or reinjury, and exhibit hypervigilance towards bodily sensations and pain [11]. This fear of movement can profoundly impact an individual’s quality of life and upper limb functional capabilities [10,11] and may significantly limit an individual’s activities [12,13], eventually restricting participation in various daily tasks. Previous studies have shown that elevated baseline kinesiophobia scores are associated with poorer outcomes related to pain, proprioception, and functional performance [14,15].

Instead, early active exercises are considered essential in the treatment of hand injuries [16,17], yet the level of kinesiophobia could directly influence patients’ adherence to these exercise regimens. Categorizing individuals by their level of kinesiophobia has provided valuable insights into how this fear affects rehabilitation and recovery outcomes [18].

The role of psychosocial factors in musculoskeletal pain is well documented [11]. Pain catastrophizing is characterized by an exaggerated perception of pain accompanied by excessive rumination, magnification, or pessimism and has been linked to chronic post-surgical pain. There is a stronger occurrence of pain catastrophizing observed following orthopedic surgeries compared with other types of surgeries [19]. Both pain catastrophizing and kinesiophobia have been associated with increased pain and disability in the shoulder. However, the findings regarding the relationship between pain catastrophizing and kinesiophobia are inconclusive [20].

Recovery after a wrist/hand injury requires a multifactorial approach to ensure optimization of an individual’s functional performance. Previous studies have shown that individuals with higher levels of kinesiophobia and greater pain have increased errors with Joint Position Sense (JPS) testing after wrist/hand injuries that involve wrist immobilization [21]. Due to the avoidance of physical exercise or movement, kinesiophobia may contribute to a deterioration of functional ability, leading to decreased mobility and chronic pain [22]. Patients with tendon repairs can develop kinesiophobia, which may contribute to difficulty when starting to re-use their hand for activities of daily living [23]. Although patients with high levels of kinesiophobia reported a higher disability level than their counterparts [23], there is no conclusive evidence regarding how kinesiophobia impacts functional performance among individuals who suffered a wrist/hand injury that required immobilization [23].

The objectives of this study are (1) to evaluate the relationship between kinesiophobia, pain catastrophizing, the *Quick*DASH, and the Patient-Rated Wrist Evaluation and (2) to evaluate the data regarding the influence that kinesiophobia may have on upper limb function after wrist/hand immobilization. We hypothesize that kinesiophobia is significantly associated with pain catastrophizing and upper limb function. The findings of this study may offer essential insights into the interactions among kinesiophobia, pain catastrophizing, and functional performance as well as the clinical management of kinesiophobia in patients that require wrist immobilization after a wrist/hand injury. By addressing the immediate post-immobilization phase and the predictive role of baseline kinesiophobia, our study will provide clinically relevant insights into functional recovery pathways that are underexplored in previous investigations.

## 2. Materials and Methods

### 2.1. Study Design and Participants

From November 2023 through March 2024, we recruited patients from different hospital centers with wrist and/or hand injuries that required immobilization after the injury had received either surgical or conservative treatment. Patients were included if they were over 18 years of age and had agreed to participate and if they had the ability to understand the informed consent and the self-administered scales. Participants were excluded if they had suffered a previous injury of the same hand, had incurred severe concomitant injuries, or were unable to provide informed consent.

All procedures were in accordance with the ethical standards of the local institutional ethics committee and with the Declaration of Helsinki of 1975, as revised in 2008 [24]. Appropriate informed consent was obtained from all participants prior to enrollment in the study.

G-power 3.1 software (Universities, Düsseldorf, Germany) [25] was used to calculate the sample size for the study. The primary outcome of this study was the score (mean and standard deviation) on the Tampa Scale of Kinesiophobia (TSK), which was used to estimate the sample size [26]. To calculate an exact sample size, we conducted a power analysis considering the expected effect size: the number of predictors in the regression model and the desired power level (0.80) and significance level (0.05). Based on our calculations, it was determined that approximately 80 participants would be an optimal sample size to ensure more robust results and statistical power.

This study adhered to the STROBE checklist [27], which provides general recommendations for descriptive observational studies and research on associations between exposure variables and health outcomes.

### 2.2. Outcome Assessment and Measures

The first study assessment took place between 5 and 10 days after the immobilization phase and during the initial hand therapy evaluation. The initial assessment included the collection of demographic information and the participant completion of the Tampa Scale of Kinesiophobia (TSK), the Pain Catastrophizing Scale (PCS), the *Quick*DASH questionnaire, and the Patient-Rated Wrist Evaluation (PRWE). Informed consent was obtained at the time of the data collection. A second round of data collection occurred 3 months after the immobilization phase.

#### 2.2.1. Tampa Scale of Kinesiophobia (TSK)

Fear of movement, or kinesiophobia, was assessed using the Tampa Scale of Kinesiophobia (TSK). The TSK consists of 17 statements, such as “I am afraid I might injure myself if I exercise”, where participants rate their level of agreement (strongly disagree, somewhat disagree, somewhat agree, and strongly agree; Scores range from 17 to 68, with higher scores indicating greater fear of pain, movement, and injury. A cutoff score of 36 is used to determine the presence of kinesiophobia [28]. The TSK is a reliable (r = 0.78) and valid measure of the fear of movement, demonstrating good internal consistency (Cronbach’s alpha = 0.80) [20].

#### 2.2.2. Pain Catastrophizing Scale (PCS)

The PCS is one of the most commonly used tools to evaluate pain catastrophizing and includes 13 items scored on a 5-point Likert scale from 0 (never) to 4 (always), with dimensions of rumination, magnification, and helplessness. Scores close to 13 indicate low levels of catastrophizing, while higher scores indicate elevated catastrophizing [29]. The Spanish version of the PCS showed appropriate internal consistency (Cronbach’s alpha = 0.79), test–retest reliability (intraclass correlation coefficient = 0.84), and sensitivity to change (effect size < or = 2) [30].

#### 2.2.3. *Quick*DASH Questionnaire

The shortened form of the Disability of the Arm, Shoulder, and Hand (DASH) questionnaire—the *Quick*DASH—was used to measure upper extremity functional performance [31]. This tool consists of 11 items and a total score ranging from 0 to 100, where 0 indicates no limitation and 100 suggests full disability. Eight questions inquire about the patient’s ability to perform certain daily activities [32]. The *Quick*DASH is as effective as the full DASH in detecting meaningful change or “responsiveness” in this patient population.

#### 2.2.4. Patient-Rated Wrist Evaluation (PRWE)

The Patient-Rated Wrist Evaluation (PRWE) was developed to assess pain in the wrist joint and functional difficulties in activities of daily living resulting from injuries affecting the wrist joint area [33]. The PRWE is a 15-item questionnaire designed to measure a patient’s wrist pain and disability. It consists of two subscales (pain and function) and has a score range from 0 (no disability) to 100 (severe disability). The Spanish version of the PRWE showed high internal consistency, test–retest reliability, and good construct validity (Cronbach’s alpha = 0.98; ICC = 0.94) [34].

### 2.3. Data Analysis

Data were analyzed using JAMOVI Desktop software (version 2.3.28 solid). Descriptive statistics, including the mean and standard deviation (SD), were calculated for all participants, and normality of the data was confirmed via the Shapiro–Wilk test. Demographic characteristics were summarized using descriptive analysis. For the primary aim, Pearson’s correlation coefficient (r) was employed to assess the linear relationship between kinesiophobia, pain catastrophizing, the PRWE, and upper limb function measured with the *Quick*DASH. Correlation coefficients were interpreted as follows: 0 to 0.3 indicated a weak relationship; 0.4 to 0.6 indicated a moderate relationship; and 0.7 to 1.0 indicated a strong relationship [35]. For the second objective, a *p*-value of ≤0.05 was used to determine statistical significance in the linear regression analysis, which was performed to assess whether kinesiophobia might interfere with upper limb function in participants who had required wrist and hand immobilization following a traumatic injury.

## 3. Results

### 3.1. Characteristics of the Participants

In the initial sample, 71 participants were recruited. Subsequently, a total of 64 participants were included in the analysis study, comprising 40 women (62.5%) and 24 men (37.5%). The mean age of the participants was 42.87 years (SD = 15.09), reflecting a wide age range. Regarding hand dominance, 47 participants (73.4%) identified as right-handed; of these, 40 (62.5%) had sustained injuries to their dominant hand.

Treatment approaches varied, with 34 participants having received conservative treatment and 30 having undergone surgical intervention. The average immobilization period was 13.78 days (SD = 12.22), indicating considerable variation across patients. The most common injury was a distal radius fracture, reported in 17 cases. Tendon injuries and carpal fractures were observed in 11 participants, while ligament injuries were noted in 10 cases. Phalangeal fractures and neuropathies were the least common diagnoses, with 8 and 7 cases, respectively.

The *Quick*DASH questionnaire showed a mean score of 45.53 with a standard deviation of 19.37, and the PRWE showed a mean score of 44.2 (SD 22.1), indicating a moderate perceived disability in the sample at baseline. The TSK yielded a mean score of 23.04 with a standard deviation of 7.05. The PCS had a mean score of 24.9 with a standard deviation of 12.3. These scores indicated a moderate level of fear of movement and catastrophizing, respectively, among the participants at baseline (Table 1).

### 3.2. Associations Between Kinesiophobia, Pain Catastrophizing, and Upper Limb Function

Pearson’s correlation coefficients were calculated to assess the relationships between variables at baseline. Significant positive correlations were found between the TSK and the *Quick*DASH (*r* = 0.848, *p* < 0.001) as well as the TSK and the PCS error (*r* = 0.521, *p* < 0.001). These correlations indicate that higher levels of kinesiophobia are associated with increased pain catastrophizing and greater upper limb dysfunction (Table 2).

### 3.3. Regression Analysis of Predictors on Outcome Variable

A multiple regression analysis was conducted to examine whether kinesiophobia could predict upper limb function. The regression model explains 30.4% of the variance in upper limb function, suggesting that the PRWE, the Pain Catastrophizing Scale, and the *Quick*DASH questionnaire are important in predicting dysfunction (Table 3).

In Model 2, without pain catastrophizing, kinesiophobia becomes a significant predictor (*p* = 0.045); however, its effect size is moderate compared with the *Quick*DASH and the PRWE, which both remain strong and significant predictors (R² = 0.250). Table 4 summarizes this model.

## 4. Discussion

Recent studies reinforce the importance of specialized research into wrist/hand injuries, particularly in post-immobilization recovery, highlighting critical areas such as stiffness, reduced range of motion, and functional impairments that emerge after immobilization [36]. We examine the relationship between kinesiophobia, pain catastrophizing, the *Quick*DASH, and the Patient-Rated Wrist Evaluation (PRWE) in patients recovering from wrist/hand injuries immediately after immobilization—a critical yet underexplored phase in rehabilitation. While prior research has investigated psychological factors in upper extremity disability [37], carpal tunnel syndrome [38], complex regional pain syndrome [39], and distal radius fractures [40], these studies do not address the unique challenges of the post-immobilization period or the broader scope of wrist/hand injuries. Our study provides new insights into the role of psychological factors in predicting functional recovery, offering valuable guidance for clinicians to optimize post-immobilization care.

Kinesiophobia is a frequent psychological condition characterized by a fear of movement that can develop after an injury [41]. The fear of movement can hinder rehabilitation [32,33], exacerbate pain, and contribute to a cycle of disability [34,35]. More studies have investigated the relationship between fear of movement and chronic pain in the upper limb, finding a positive relationship between pain levels and fear of movement [3,42]. For example, Cantero-Téllez et al. [21] explored the relationship between kinesiophobia, pain intensity, and joint position sense in distal radius fractures but focused on proprioception rather than functional recovery. Bartlett and Farnsworth [43] investigated kinesiophobia’s impact on perceived disability in upper extremity injuries but did not specifically examine the post-immobilization period or functional outcomes. Similarly, Meugnot et al. [44] analyzed neural and sensorimotor changes after short-term immobilization but did not consider psychological predictors like kinesiophobia. While our study finds a positive correlation between kinesiophobia and perceived disability, the regression analysis suggests it is not a significant independent predictor when other factors like pain catastrophizing are considered. This aligns with findings by Crombez et al. [45] and Leeuw et al. [46], who emphasize that fear of movement contributes to disability indirectly, often through mechanisms like avoidance behaviors and catastrophic thinking. The moderate effect size in the absence of pain catastrophizing underscores the intricate interplay of psychological factors, suggesting the need for more comprehensive investigations into these complex relationships.

Likewise, Lentz et al. noted that fear of pain was a minor contributor to function in patients with shoulder-related disorders [47]. Based on our findings and in concordance with prior research, greater baseline kinesiophobia and pain catastrophizing have been identified as predictors of more severe post-operative pain [42] and may also predict significant disability in individuals with various upper extremity conditions [37] The stronger predictive value of pain catastrophizing compared with kinesiophobia aligns with Vlaeyen and Linton’s fear-avoidance model [48,49], which posits that catastrophic thinking amplifies pain perception, contributing to a cycle of disability and psychological distress. In our model, if we excluded pain catastrophizing, kinesiophobia became a significant predictor (*p* = 0.045); however, its effect size is moderate, while the *Quick*DASH and the PRWE remain strong and significant predictors. The *Quick*DASH and the PRWE are valuable tools for assessing upper limb functional performance, and the baseline *Quick*DASH score had the largest impact on predicting upper limb function according to our model. This could be because the *Quick*DASH is more effective in assessing upper limb function because it comprehensively addresses a wide range of issues, is good at detecting change over time, focuses on the patient’s personal experience, and considers psychological aspects like fear of movement [50]. The inclusion of these variables in our model is one of the strengths of our study, as few investigations consider the specific functional variables of the upper limb in their analyses.

It is not possible to determine whether fear of movement leads to increased pain as a result of reduced joint mobility [3], or if it may be related to strict post-operative instructions given to the patient when they require immobilization of the injured hand or wrist [23,51]. But, the significant positive correlation between kinesiophobia and upper limb functional performance (TSK and *Quick*DASH, r = 0.848, *p* < 0.001) suggests that a higher level of fear of movement is strongly associated with greater perceived disability in daily tasks. The lack of significance for kinesiophobia as a standalone predictor (*p* = 0.06) could imply that the fear of movement may not directly influence disability but, rather, operates through its interaction with other psychological factors, such as pain catastrophizing, which has been identified as a more robust predictor of disability in chronic pain populations [41,42]. Instead, only a few hand therapists routinely assess psychological status during evaluations [52]. Our findings underscore the need for integrating psychological assessments into routine rehabilitation practices and incorporating cognitive behavioral therapy (CBT) and graded exposure techniques, which have been shown to reduce maladaptive thinking and enhance recovery [45,46,48].

Several limitations should be considered in our results. Given that our study involves examining correlations, detecting moderate to strong correlations, and considering other practical factors, a sample size of approximately 80 participants would have been more optimal to ensure more robust results and statistical power. Additionally, the sample’s characteristics, including injury type, severity, and demographic factors, may restrict the generalizability of our findings. Future research should include a more diverse population to improve external validity and determine if these relationships hold across different subgroups.

Longitudinal studies could provide clarity on whether early interventions targeting fear of movement influence long-term functional recovery. Furthermore, examining the effectiveness of tailored psychological interventions across different upper limb conditions could yield valuable insights for rehabilitation strategies. By addressing the immediate post-immobilization phase and the predictive role of baseline kinesiophobia, our study fills a distinct gap, providing clinically relevant insights into functional recovery pathways that are underexplored in these references.

## 5. Conclusions

In summary, this study reinforces the association between kinesiophobia and upper limb dysfunction but suggests that kinesiophobia alone may not be a significant predictor of functional outcomes when other factors like pain catastrophizing and injury-specific severity are considered. Future research should aim to further delineate the interaction between psychological and physical variables in upper limb rehabilitation and explore targeted interventions to reduce fear of movement and catastrophic thinking in this patient population.

## Figures and Tables

**Table 1:** Descriptive analysis.

*Descriptive Analysis (N = 64)*	
Variable	Mean (SD)
Age	42.87 (15.09)
Time of immobilization (Days)	18.3 (12.22)
TSK baseline	23.04 (7.05)
PCS baseline *Quick*DASH baseline PRWE baseline	24.9 (12.32) 45.5 (19.4) 44.2 (22.1)

Note. Descriptive analysis (N = 64); TSK (Tampa Scale of Kinesiophobia); PCS (Pain Catastrophizing Scale); *Quick*DASH (short version of Disability of the Arm, Shoulder, and Hand—DASH); and PRWE (Patient-Rated Wrist Evaluation).

**Table 2:** Pearson’s correlation coefficients considering TSK, *Quick*DASH, PRWE, and PCS.

Variables	r	*p*	N
TSK–*Quick*DASH TSK–PRWE	0.848 ** 0.852 **	<0.001 <0.001	64 64
TSK–PCS	0.521 **	<0.001	64
PCS–*Quick*DASH	0.798 **	<0.000	64

Note. TSK = Tampa Scale of Kinesiophobia; *Quick*DASH = short version of Disability of the Arm, Shoulder, and Hand—DASH; and PCS = Pain Catastrophizing Scale. ** The correlation is significant at 0.01.

**Table 3:** Model 1. Regression analysis using TSK, PRWE, PCS, and *Quick*DASH.

Predictor	B	Standard Error (SE)	T-Value	*p*-Value
TSK PRWE	0.184 0.530	0.095 0.145	1.92 3.86	0.06 <0.001
PCS	0.340	0.152	2.30	0.02
*Quick*DASH	0.612	0.098	6.22	<0.001
R^2^	0.304			
Adjusted R^2^	0.292			
F-value	8.74			

Note. TSK = Tampa Scale of Kinesiophobia; PRWE = Patient-Rated Wrist Evaluation; PCS = Pain Catastrophizing Scale; *Quick*DASH = short version of Disability of the Arm, Shoulder, and Hand—DASH; B = unstandardized coefficient; and SE = standard error. The model explains 30.4% of the variance in upper limb function.

**Table 4:** Model 2. Regression analysis using TSK, PRWE, and *Quick*DASH.

Predictor	B	Standard Error (SE)	T-Value	*p*-Value
TSK PRWE	0.220 0.470	0.107 0.140	2.06 3.36	0.045 <0.001
*Quick*DASH	0.610	0.098	6.22	<0.001
R^2^	0.250			
Adjusted R^2^	0.235			
F-value	10.17			

Note. TSK = Tampa Scale of Kinesiophobia; PRWE = Patient-Rated Wrist Evaluation; *Quick*DASH = short version of Disability of the Arm, Shoulder, and Hand—DASH; B = unstandardized coefficient; and SE = standard error. The model explains 25% of the variance in upper limb function.

## Data Availability

No will share research data.
